# Repurposing Sigma-1 Receptor-Targeting Drugs for Therapeutic Advances in Neurodegenerative Disorders

**DOI:** 10.3390/ph18050700

**Published:** 2025-05-09

**Authors:** Kiarash Eskandari, Sara-Maude Bélanger, Véronik Lachance, Saïd Kourrich

**Affiliations:** 1 Département des Sciences Biologiques, Université du Québec à Montréal, 141 Avenue du Président-Kennedy, Montreal, QC H2X 3X8, Canada; eskandari.kiarash@uqam.ca (K.E.); belanger.sara-maude@courrier.uqam.ca (S.-M.B.); lachance.veronik@uqam.ca (V.L.); 2 Centre d’Excellence en Recherche sur les Maladies Orphelines-Fondation Courtois, Pavillon des Sciences Biologiques, Université du Québec à Montréal, 141 Avenue du Président-Kennedy, Montreal, QC H2X 3Y7, Canada; 3 Center for Studies in Behavioral Neurobiology, Concordia University, Montreal, QC H4B 1R6, Canada

**Keywords:** drug repurposing, neurodegenerative diseases, neuroprotection, sigma-1 receptor (S1R)

## Abstract

Neurodegenerative disorders, such as Alzheimer’s, Parkinson’s, and Huntington’s disease, due to their multifaced and complicated nature, remain uncurable and impose substantial financial and human burdens on society. Therefore, developing new innovative therapeutic strategies is vital. In this context, drug repurposing has emerged as a promising avenue to expedite the development of treatments for these challenging conditions. One particularly compelling target in this regard is the chaperone protein sigma-1 receptor (S1R), which has garnered significant attention for its neuroprotective properties. Interestingly, several medications, including fluvoxamine (an antidepressant), dextromethorphan (a cough suppressant), and amantadine (an antiviral), which were initially developed for unrelated indications, have shown encouraging results in neurodegenerative therapy through S1R activation. These findings suggest that existing drugs in pharmacopeias can play an essential role in alleviating neurodegenerative symptoms by modulating S1R, thereby offering a faster route and cost-effective path to clinical applications compared to the de novo development of entirely new compounds. Furthermore, as a synergistic benefit, combining S1R-targeting drugs with other therapeutic agents may also improve treatment efficacy. In this review, we highlight key repurposed drugs targeting S1R and explore their mechanisms of action, shedding light on their emerging therapeutic potential in the fight against neurodegeneration.

## 1. Introduction

Neurodegenerative diseases are a group of diverse and complex conditions that originate in distinct parts of the nervous system and lead to progressive neuronal loss. A common hallmark of these disorders is the aggregation and accumulation of misfolded proteins, which play a critical role in the disease pathogenesis. Notably, β-amyloid (Aβ) plaques and hyperphosphorylated tau aggregates characterize Alzheimer’s disease (AD), while alpha-synuclein accumulation is a key feature of Parkinson’s disease (PD). Similarly, mutant huntingtin protein drives Huntington’s disease (HD), and TAR DNA-binding protein (TDP)-43 aggregates are implicated in Amyotrophic Lateral Sclerosis (ALS) [1,2,3,4]. Beyond protein misfolding, these diseases share several pathobiological mechanisms, such as mitochondrial dysfunction, increased oxidative stress, and chronic neuroinflammation. Collectively, these processes exacerbate neuronal damage and the pathological progression of the disorders.

Although various therapies and interventions are available to manage symptoms, a definitive cure remains elusive due to the heterogeneous nature of these diseases [5]. This emphasizes the urgent need for the development of novel treatments that not only relieve symptoms but also address the root problem of neurodegeneration [6]. However, drug development is an inherently high-risk, high-cost, and time-consuming process, spanning from target identification to post-market surveillance [7]. The repurposing, i.e., the strategic retargeting of existing therapies for new disease indications, is an efficient and cost-effective alternative, significantly reducing both time and financial burden related with drug development [8]. Unlike traditional drug discovery, many early stages, such as target identification, compound screening and optimization, and preclinical testing, can be bypassed in drug repurposing, as these compounds have successfully undergone rigorous clinical evaluation. It is essential to mention that the development of a new medicine usually requires 10 to 15 years and costs between USD 2 to USD 3 billion, whereas repurposing an existing drug can shorten this timeline to 3 to 7 years and reduce costs to approximately USD 300 million [9,10,11].

The sigma-1 receptor (S1R) is a chaperone protein found in the endoplasmic reticulum (ER). Activation of S1R elicits potent neuroprotective effects and inhibits neurodegeneration via multiple mechanisms, including regulating calcium homeostasis, promoting mitochondrial functions, decreasing ER stress, and reducing inflammatory responses [12,13]. Beyond its neuroprotective role, S1R activation can modulate neurotrophic factors, which are essential for restoring lost functions and stimulating neuronal plasticity. Given these benefits, S1R has recently attracted significant interest as a promising target for neurodegenerative disease research, with studies exploring its therapeutic potential in AD, PD, ALS, and HD [12,13]. Importantly, drugs such as Anavex2-73 have demonstrated encouraging results in clinical trials, further reinforcing the therapeutic promise of S1R-targeting compounds [14].

Considering the time and costs associated with the discovery of novel therapeutic agents, and the well-documented protective role of S1R, this review aims to highlight the effectiveness of available drugs that target S1R as a potential treatment for neurodegenerative diseases—particularly those lacking viable therapies.

## 2. Sigma-1 Receptor: Biology and Mechanism of Action

### 2.1. S1R as a Chaperone Protein and Inter-Organelle Signaling Modulator

S1R is a 223-amino-acid (26 kDa) protein coded by the SIGMAR1 gene primarily localized at the ER membrane within cholesterol-rich areas known as the mitochondrial-associated membrane (MAM) [15,16]. In addition to some peripheral organs, including the heart, liver, and eyes, S1R is highly abundant in the central nervous system (CNS), including neurons, astrocytes, oligodendrocytes, and microglia, with particularly high concentrations in the limbic system, and motor control neurons [17,18,19].

Functionally, S1R acts as an intracellular receptor with a chaperone protein function. In its resting state, S1R is bound via its carboxy-terminal region to glucose-related protein 78/binding immunoglobulin protein (BiP), another ER-resident chaperone protein. This complex prevents S1R from engaging with other cellular proteins or signaling pathways [20,21]. However, upon agonist binding or in response to ER stressors—such as the accumulation of misfolded proteins, oxidative stress, and calcium imbalance—BiP dissociates from S1R, triggering its activation. Once induced, S1R facilitates communication between the ER and mitochondria and can also translocate from the MAMs to the plasma membrane, cytoplasmic membrane systems, and nuclear envelope. As a result, S1R acts as a key inter-organelle signaling modulator that directly or indirectly regulates various functional proteins and ion channels [22,23,24].

### 2.2. Mitochondrial Function

Mitochondria, often described as the powerhouses of the cell, are essential cellular component responsible for ATP production and generating reactive oxygen species (ROS). In these organelles, ATP is produced through a highly orchestrated series of biochemical reactions comprising the tricarboxylic acid cycle (TCA) and the electron transport chain (ETC), collectively constituting the process of oxidative phosphorylation. Mitochondria simultaneously serve as the leading site for producing ROS, which are generated as inevitable byproducts during the process of electron transfer along the respiratory chain complexes [25]. The disruption of mitochondrial homeostasis has been consistently linked to progressive oxidative damage and general energetic failure [26]. The in vitro and in vivo experiments of Leuner et al. support that mitochondria-derived ROS enhance the processing of the Aβ precursor protein (AβPP) to Aβ [27]. Ballard et al. also showed that exposure to 1-methyl-4-phenyl-1,2,3,6-tetrahydropyridine (MTPT), a complex I inhibitor in ETC, generates permanent human parkinsonism with extensive degeneration of nigral neurons [28].

**Figure 1:**
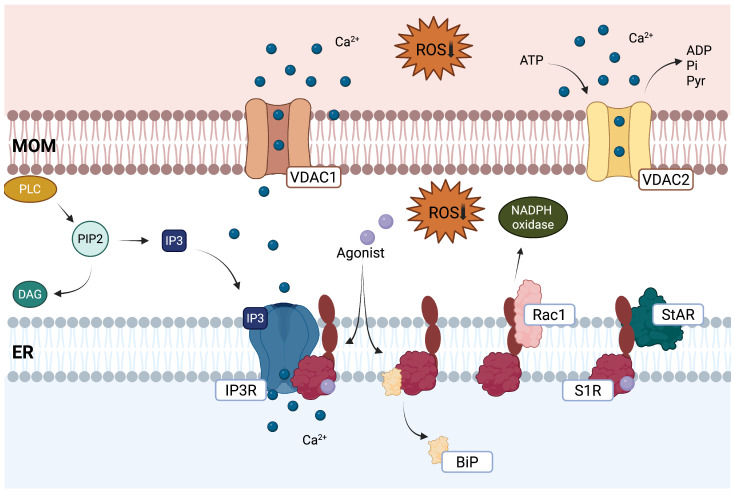
Role of S1R in regulating mitochondrial function. At the MAMs, S1R modulates calcium signaling by interacting with the IP3R receptor, facilitating the transfer of calcium from the ER to the mitochondria via the VDAC1 channel. S1R interacts with the StAR protein and the VDAC2 channel, thus promoting transporting key energy metabolites, including pyruvate, ATP, ADP, and phosphate. S1R also modulates mitochondrial oxidative stress by acting on Rac1 and NADPH oxidase signalling. By inhibiting excessive Rac1 activation, S1R reduces NADPH oxidase activity, limiting ROS production and protecting mitochondria from oxidative stress. Created in BioRender. https://BioRender.com/s34d665 (accessed on 15 April 2025).

At the MAM, S1R stabilizes the inositol triphosphate receptor (IP3R) and promotes the controlled release of calcium from the ER into the mitochondria [20]. Activation of S1R also stimulates phospholipase C (PLC), leading to an increase in inositol triphosphate (IP3), which, through activation of IP3R, also increase calcium release into the mitochondria and cytosol [15,29]. Given that calcium serves as an essential cofactor for numerous enzymes involved in the TCA cycle and ETC activity, S1R plays a pivotal role in modulating energy production [30]. Goguadze et al. showed that S1R increased the activity of complex I in a calcium-dependent and S1R antagonist-sensitive manner. S1R activation also attenuated Aβ-induced complex I and IV dysfunctions [31]. In addition, it has been shown that S1R forms complexes with the steroidogenic acute regulatory protein (StAR) and regulates the voltage-gated anion channel 2 (VDAC2), which is essential in transporting energy metabolites, including succinate, pyruvate, NADH, ATP, ADP, and phosphate [32,33]. It should be noted that by enhancing oxidative phosphorylation, S1R triggers moderate ROS formation [31]. Additionally, S1R directly interacts with Ras-related C3 botulinum toxin substrate 1 (Rac1-GTPase), which promotes the activity of NADPH oxidase, an alternative source of cellular and extracellular ROS [34]. Rather than representing a detrimental effect, this controlled ROS generation appears to function as a physiological signal that ultimately strengthens cellular antioxidant defenses. The mild ROS generation induced by S1R activation, ultimately leads to upregulation of the antioxidant response element (ARE), stimulating increased transcription of crucial protective genes, including NAD(P)H quinone oxidoreductase 1 (NQO1) and superoxide dismutase 1 (SOD1) [35]. S1Rs, through their strategic positioning at the MAMs, play a multifaceted role in fine-tuning mitochondrial homeostasis. This complex interplay provides valuable insights into both normal cellular physiology and the pathogenesis of neurodegenerative disorders. The capacity of S1R to simultaneously enhance energy production while triggering protective antioxidant mechanisms presents possibilities for therapeutic interventions targeting mitochondrial dysfunction in conditions such as AD and PD (Figure 1).

### 2.3. Endoplasmic Reticulum Stress

The ER is responsible for protein synthesis, folding, and post-translational modification. However, various physiological and pathological conditions can disrupt ER function, promoting the accumulation of misfolded or unfolded proteins within its lumen [36]. This disruption, referred to as ER stress, triggers a highly conserved signaling network known as the unfolded protein response (UPR). The UPR plays a dual role: it not only initiates protective or adaptive mechanisms to restore ER homeostasis but can also trigger apoptosis if the stress remains unresolved [36,37]. The three primary transducers of the UPR are PERK (PKR-like ER kinase), IRE1 (inositol-requiring 1), and ATF6 (activating transcription factor 6). PERK phosphorylates eukaryotic initiation factor 2 (eIF2α) and the nuclear factor (erythroid-derived 2)-like 2 (Nrf2), which inhibit general protein synthesis (to minimize the load of new proteins entering the ER) and activate genes that encode detoxification enzymes (to counteract oxidative stress), respectively [38]. PERK activates ATF4, which is important in autophagy-related gene regulation and adaptative stress response [39,40]. IRE1 mediates the splicing and activation of the X-box-binding protein 1 (XBP-1). It induces the expression of ER-associated degradation (ERAD) components, chaperones, and proteins involved in lipogenesis, promoting cell resilience under stress conditions. [41,42]. ER stress also induces ATF6 translocation to the Golgi apparatus, where it is cleaved into an active form that upregulates target genes that encode ER chaperones, ERAD constituents, and XBP1 [42,43]. This enhances the cell’s capacity to manage misfolded proteins. While the UPR initially aims to restore homeostasis, a chronic or excessive UPR activation pathway can shift cellular response toward apoptosis. A critical mediator of this transition is CHOP (C/EBP homologous protein), which is upregulated through the mentioned pathways. CHOP induces apoptosis by suppressing anti-apoptotic factor B cell lymphoma-2 (BCL-2) family members, such as BCL-2-associated X protein (BAX) and BCL-2 antagonist/killer (BAK). This inhibition enhances mitochondrial dysfunction and cell death [44,45,46].

**Figure 2:**
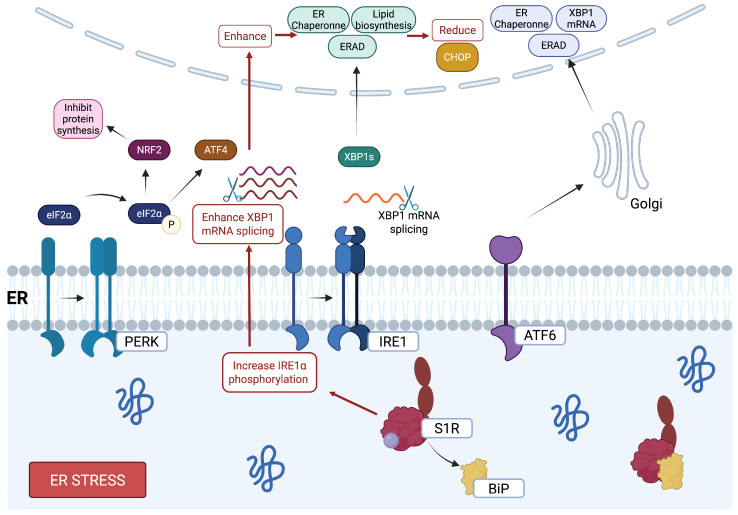
S1R modulation of ER stress response and UPR pathways. At the level of ER stress, S1R interacts with three ER stress transmembrane sensors involved in UPR: IRE1, PERK, and ATF6. This activation promotes the increased expression of XBP1 mRNA, ERAD, ER chaperones, and enzymes involved in lipid biosynthesis, while reducing the expression of CHOP, a pro-apoptotic factor. S1R can modulates the PERK pathway, which plays a crucial role in balancing protein synthesis and cellular adaptation to stress. By regulating eIF2α phosphorylation, S1R helps attenuate global protein translation while selectively promoting the expression of stress-response genes, such as ATF4. Created in BioRender. https://BioRender.com/y50m142 (accessed on 15 April 2025).

Several studies have demonstrated that S1R activation influences UPR signaling in ways that mitigate ER stress-induced apoptosis. It has been shown that S1R activation increases IRE1α phosphorylation, leading to enhanced splicing of XBP1 mRNA, which enhances gene expression that supports UPR and reduces the expression of CHOP, protecting cells against ER stress-induced damage [47,48,49]. Moreover, upon stress induction, S1R expression is upregulated via the PERK/eIF2α/ATF4 pathway, and its overexpression suppresses the excessive activation of key UPR stressors, such as PERK and ATF6, further reducing the expression of CHOP and inhibiting apoptosis [20,46,50]. By modulating UPR signaling pathways, S1R limits the pro-apoptotic effects associated with prolonged ER stress. This includes downregulating CHOP and preserving mitochondrial integrity by maintaining BCL-2 family balance (Figure 2). Altogether, these protective properties position S1R, again, as a promising therapeutic for diseases associated with chronic ER stress and dysregulated UPR activity, such as neurodegenerative disorders.

### 2.4. Autophagy

**Figure 3:**
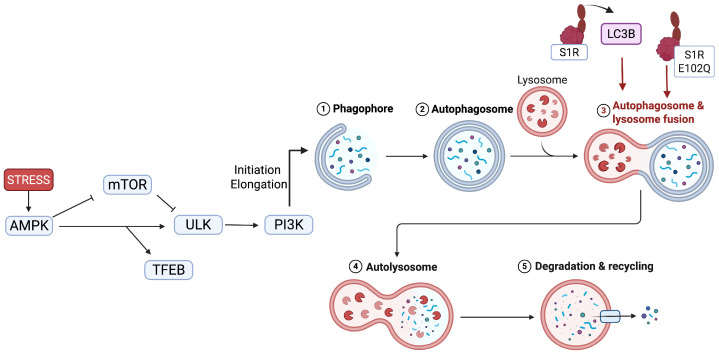
Impact of S1R in the initiation and maturation of autophagy. S1R plays a role in regulating autophagy by influencing both autophagosome initiation and maturation. S1R modulates the expression and activation of LC3B, a key marker of autophagosome formation. Under conditions of cellular stress, AMPK is activated, leading to the inhibition of mTOR, a major suppressor of autophagy. This inhibition releases the repression on the ULK complex (initiating autophagosome formation) that activates PI3K (role in autophagosome–lysosome fusion). The activation of AMPK influences TFEB, a key transcription factor regulating lysosomal biogenesis and autophagic genes, reinforcing the autophagic flux. Created in BioRender. https://BioRender.com/g58q783 (accessed on 15 April 2025).

Autophagy is an intracellular lysosomal-dependent degradation system responsible for eliminating damaged or obsolete cellular components. Two key signaling complexes, ULK (ULK1/2, FIP200, and ATG13) and PI3K (Beclin1, Vps34, VPS15, and ATG14), are required for initiating autophagosome formation. The nutrient and energy sensor mammalian target of rapamycin complex1 (mTORC1) and AMP-activated kinase (AMPK) signal directly to the ULK complex and influence its activity [51]. Additionally, both mTORC1 and AMPK activate transcription factor EB (TFEB), a master transcriptional regulator of autophagy, to enhance lysosomal function and autophagy flux [52,53]. In most neurodegenerative diseases, including ALS, PD, and AD, the accumulation of misfolded proteins due to autophagy impairment is notable [54,55]. Reduced Beclin1 expression and other ATG-related genes have been observed in the brains of AD-affected patients, and this impairment appears early in the disease course [56,57]. Misfolded proteins can also bind directly to key autophagy components, such as Beclin1 and others, and disrupt the formation of autophagosomes. Furthermore, the fusion of autophagosomes with lysosome can be hindered by aggregated proteins, further complicating cellular debris clearance [58]. In both AD and PD, key proteins involved in mitophagy, such as PINK1/Parkin, are impaired [59,60]. TFEB deficiency is also commonly observed in neurodegenerative diseases, leading to the accumulation of toxic proteins and dysfunctional organelles [61,62]. Current evidence suggests that S1R plays a pivotal role in autophagy regulation. A genetic variant of S1R (p.E102Q) increases susceptibility to juvenile ALS and seems to influence autophagosome fusion with lysosomes [63]. In one study, the knockdown of S1R in dopaminergic cells led to decreased levels of ULK1 and other proteins that promote autophagy, as well as the reduced recruitment and stabilization of the PINK1/Parkin pathway in mitochondria [64]. Another study confirmed S1R’s involvement in mitophagy through interactions with ATG14, STX17, and VAMP8 [65]. Moreover, Wang et al. recently demonstrated that the activation of S1R enhances the translocation of the transcription factor TFEB into the nucleus and stimulates autophagy [66]. Given these findings, stimulating S1R appears to be a beneficial strategy in treating neurodegenerative diseases, as it promotes the autophagy mechanism and facilitates the degradation of dysfunctional cellular proteins and organelles (Figure 3).

### 2.5. Neuroinflammation

**Figure 4:**
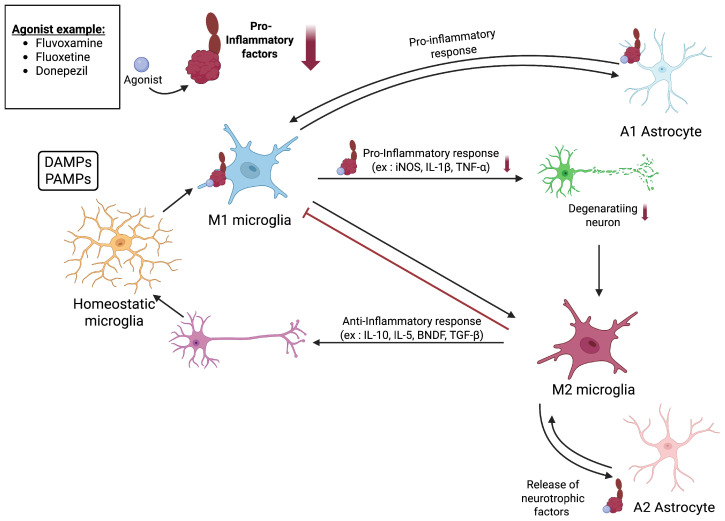
Impact of S1R activation by an agonist on neuroinflammation. S1R activation by an agonist influences neuroinflammation by modulating both microglia and astrocytes. Microglia are activated in response to DAMPs and PAMPs, which can trigger their polarization toward a pro-inflammatory M1 phenotype, promoting the production of inflammatory cytokines and contributing to neuronal degeneration. In contrast, anti-inflammatory M2 microglia counterbalance this activation by reducing neuroinflammation and restoring homeostasis. Astrocytes contribute to neuroinflammation through distinct reactive states. A1 astrocytes, induced by pro-inflammatory signals, exacerbate neurotoxicity and neurodegeneration, whereas A2 astrocytes exhibit neuroprotective properties by promoting neuronal survival and tissue repair. S1R activation may help suppress A1-astrocyte formation while promoting A2 astrocytes, further reinforcing the anti-inflammatory environment and neuroprotection. Created in BioRender. https://BioRender.com/v48v603 (accessed on 15 April 2025).

Microglia, the resident macrophages in the brain and spinal cord, are the primary mediators of neuroinflammation. These cells play a dual role in neurodegenerative diseases that depend on the microglia’s functional phenotypes [67,68]. Microglia can adopt either a pro-inflammatory M1 or an anti-inflammatory M2 phenotype, each contributing differently to the progression of neurodegeneration. M1-polarized microglia are characterized by the release of inflammatory cytokines, such as tumor necrosis factor (TNF)-α, interleukin (IL)-1β, and IL-6, which enhance the inflammatory response. In contrast, M2-polarized microglia secrete neurotrophic factors, including brain-derived neurotrophic factor (BDNF), which support the survival, repair, and regeneration of neurons [69,70,71,72]. TLR4 (Toll-like receptor 4) is a key player in recognizing damage-associated molecular patterns (DAMPs), such as Aβ in AD and alpha-synuclein in PD. It activates stress-induced pathways, including Nuclear Factor Kappa B (NF-κB), mitogen-activated protein kinases (MAPKs), extracellular signal-regulated kinase (ERK), and JNK, contributing to microglial M1 polarization and inflammation [73,74]. In addition to microglia, astrocytes also play a significant role in regulating neuroinflammation. Reactive astrocytes can be classified in A1 and A2 subtypes. In response to various insults, A1 reactive astrocytes proliferate and release exocytotic gliotransmitters and proinflammatory cytokines. In contrast, A2 astrocytes release neurotrophic factors such as BDNF [75,76,77]. As mentioned, neurodegenerative disorders are associated with increased levels of proinflammatory cytokines, like IL-6, TNF-α, and IL-1β. These cytokines activate astrocytes through pathways such as JAK-STAT suppression [78], leading to morphological changes and the increased expression of glial fibrillary acidic protein (GFAP). Although this activation supports the formation of glial scars to isolate the damaged areas and protect the surrounding neurons, excessive astrocyte activation may impair neuronal repair and hinder neuronal regeneration [79]. Interestingly, S1R has emerged as a key modulator of neuroinflammatory responses in both microglia and astrocytes. Its activation is reported to inhibit microglial migration and activation, thereby modulating their function. Activated S1R reduces the secretion of pro-inflammatory factors, including TNF-α, nitric oxide (NO), IL-10, and several others [80,81,82,83,84,85,86]. Although most research has focused on the M1 type, it seems that activation of S1R also promotes microglial transitions from pro-inflammatory M1 to anti-inflammatory M2 [87]. Activation of S1R in astrocytes has also been shown to reduce the secretion of pro-inflammatory cytokines, such as IL-1β, TNF-α, and iNOS [88]. These anti-inflammatory effects are thought to involve ERK and NF-kB signaling pathways, which are also implicated in microglial activation [89,90]. Furthermore, S1R has been shown to affect the Janus kinase 2 (JAK2)/STAT3 signaling pathway, preventing excessive GFAP expression and improving reactive astrogliosis [87,91,92]. In summary, by targeting S1R, microglial polarization can be shifted toward the protective M2 phenotype, excessive activation of astrocytes can be mitigated to prevent detrimental effects on neuronal repair, and the release of neurotrophic factors can be enhanced to support neuronal regeneration [88,93]. Therefore, these findings reinforce S1R’s therapeutic potential in neurodegenerative diseases (Figure 4).

### 2.6. Neurotrophic Factors

Neurotrophic factors, including BDNF, nerve growth factor (NGF), and glial cell-derived neurotrophic factor (GDNF), play a pivotal role in neuronal survival, synaptic plasticity, and injury response. These proteins bind to specific receptors, such as tyrosine receptor kinase B (TrkB), activating downstream signaling pathways like PI3K/Akt and MAPK/ERK, which promotes neuronal survival and synaptic plasticity [94,95]. However, in neurodegenerative diseases, the level or activity of these factors is often impaired, accelerating neuronal loss and disease progression. The expression of BDNF is reduced in the motor cortex and in thalamic input, which correlates with motor dysfunction and striatal atrophy, a hallmark of HD pathology [96]. Furthermore, reduced BDNF protein and mRNA levels in the neocortex and hippocampus have been associated with AD disease, underscoring its role in memory and synaptic maintenance [97]. BDNF depletion is associated with Aβ accumulation, tau phosphorylation, neuroinflammation, and neuronal apoptosis [98]. Likewise, NGF is necessary for maintaining cholinergic neurons’ integrity and survival, whose degeneration contributes to cognitive deficits in AD [99,100]. GDNF, identified initially as a survival factor for dopaminergic neurons in the midbrain, is critical for protecting these neurons from degeneration in PD [101,102]. Interestingly, S1R modulates neurotrophic factor activity through multiple interconnected mechanisms, including calcium signaling, synaptic plasticity, IRE1-XPB1 pathway activation, and growth factor synergy [103]. Indeed, S1Rs modulate calcium entry through NMDA receptors by facilitating their transport to the plasma membrane and enhancing the expression of GluN2A and GluN2B subunits [103,104,105,106]. Following this calcium influx event, CaMKIV/II (calcium/calmodulin-dependent protein kinases) activity is induced and triggers downstream pathways (ERK1/2 and mTOR) that phosphorylates the cyclic-AMP response element-binding protein (CREB). CREB activation boosts BDNF transcription, promoting synaptic plasticity and neuronal survival [107,108]. S1R localization at the MAMs stabilize IRE1, enhancing XBP1 splicing and activation, which has been shown to increase the levels of BDNF expressed within neurons [48,103,109,110]. Beyond these pathways, S1R activation also amplifies NGF-induced neurite outgrowth, epidermal growth factor (EGF)-induced neurogenesis, and GDNF-dependent repair mechanisms, thereby creating a synergistic environment for neuronal health [111,112,113,114,115]. In conclusion, S1R acts as a molecular hub that integrates calcium signaling, stress-response pathways, and growth factor activity to bolster neurotrophic support. Its ability to enhance BDNF, NGF, and GDNF signaling—while mitigating neurodegenerative deficits—positions S1R agonists as promising therapeutics for AD, PD, HD, and other disorders marked by neurotrophic factor depletion. Targeting S1R could simultaneously address synaptic dysfunction, neuronal loss, and repair mechanisms, offering a multifaceted strategy to slow or reverse disease progression.

## 3. Repurposing Existing Drugs Targeting the S1R for Neurodegenerative Diseases

Several existing medications, which were initially formulated for different indications, have exhibited some potential in targeting S1R and could be beneficial in the treatment of neurodegenerative disorders (Table 1). These drugs have a dual mechanism of action that relies not only on well-known effects but also on new signaling pathways that may be helpful in neuroprotection. The selection of these agents was informed by a comprehensive literature search across databases such as PubMed and Scopus, focusing on compounds with documented or putative S1R agonist activity, as evidenced by preclinical and/or clinical evidence. Notably, certain agents—such as amantadine and memantine—are included despite their relatively low S1R affinity (K_i_ values in the micromolar range), owing to their established clinical utility and mechanistic data implicating S1R involvement. This broader inclusion aims to provide a translationally relevant overview of S1R modulation as a therapeutic strategy in neurodegenerative diseases.

### 3.1. Fluvoxamine

Fluvoxamine (Luvox, Floxyfral, and Faverin)™ is a well-known selective serotonin reuptake inhibitor (SSRI) that increases serotonin concentrations in the brain and is most commonly prescribed for depressive disorders and obsessive-compulsive disorder [120]. However, its role as a potent S1R agonist, with a binding affinity (K_i_) of 36 nM, has expanded its therapeutic potential far beyond mood regulation [121,122]. For comparison, PRE-084, which is a highly selective S1R agonist, exhibits a K_i_ of approximately 2.2 nM [123]. Fluvoxamine’s S1R agonism drives its neuroprotective effects through multiple pathways. It promotes mitochondrial function by enhancing calcium influx into mitochondria and inducing ATP production [124]. Fluvoxamine also alleviates ER stress by upregulating S1R expression via ATF4 translation. This effect is blocked when an S1R antagonist is administered, highlighting once more the importance of S1R in fluvoxamine’s mechanism of action [125]. Additionally, fluvoxamine has shown anti-inflammatory activity by decreasing the levels of pro-inflammatory cytokines, such as IL-6, IL-1β, IL-12, and IL-8 [126,127]. In an animal model of multiple sclerosis, treatment with fluvoxamine was shown to decrease disease severity by decreasing serum levels of the pro-inflammatory cytokine IFN-γ and reducing demyelination, suggesting its potential as a therapeutic agent in multiple sclerosis (MS) [128]. In models of ALS, fluvoxamine has also been shown to stabilize proteins like nucleoporin Pom121, which facilitates the translocation of TFEB, enhancing autophagy and lysosomal function, which is crucial in the pathology of most of neurodegenerative diseases [129]. Fluvoxamine also appears to be an interesting candidate for treating neurological diseases, as it increases BDNF and NGF levels [112,122,130,131]. In a study by Moriguchi et al., it was demonstrated that stimulation of S1R by fluvoxamine increased BDNF mRNA expression, protein kinase B (Akt/PKB) phosphorylation, and neurogenesis in dentate gyrus (DG) [132]. Fluvoxamine has been shown to enhance NGF-induced neurite outgrowth. This effect was blocked by the co-administration of the selective S1R antagonist, further underscoring the role of S1R in the effects of fluvoxamine on neuronal outgrowth [133]. Importantly, fluvoxamine ameliorates AD pathology by reducing amyloid burden and tau phosphorylation [134]. In fact, Kim et al. showed that fluvoxamine reduced Aβ production by inhibiting γ-secretase activity and cognitive decline [135]. Human studies also add evidence of fluvoxamine’s significant effects on the behavioral and psychological symptoms of AD patients [136,137]. This indicates that fluvoxamine can hold potential as a candidate for repurposing in dementia treatment [135]. Fluvoxamine has also shown promise in the treatment of cognitive deficits, as demonstrated by an in vivo mouse model of phencyclidine (PCP)-induced cognitive impairment. This effect was antagonized by S1R antagonists, suggesting that S1R activation mediates the cognitive-enhancing effects of fluvoxamine [138,139]. In terms of movement disorders, in a case study by Albayrak et al., fluvoxamine was shown to be effective in reducing chorea as well as treating depressive disorder in a patient with HD [140]. Fluvoxamine’s unique duality transcends its traditional role as an antidepressant, emerging as a versatile therapeutic agent for neurodegenerative diseases by functioning both as a selective SSRI and a robust S1R agonist. By harnessing S1R activation, it targets ER stress, neuroinflammation, and protein aggregation while promoting neurotrophic support and synaptic resilience. Further research into S1R-specific mechanisms could optimize fluvoxamine’s use as a disease-modifying therapy, bridging the gap between psychiatric and neurological care.

### 3.2. Citalopram and Escitalopram

In the SSRI group, citalopram (Celexa, Cipramil, Cital)™ and escitalopram (Cipralex, Seroplex, Esipiram)™ were also shown to have significant affinity for S1R (K_i_ = 292 nM and K_i_ = 288.3 nM, respectively), suggesting that their therapeutic effects may extend beyond their serotonergic actions. It has been demonstrated that it reduces the expression of key proteins involved in ER stress response and apoptosis, including PERK, GRP78, XBP1, and CHOP, as well as caspase-12 [141,142]. Citalopram-treated cells enhance mRNA levels of autophagy-related genes, such as LC3A, LC3B, ATG5, and Beclin1, suggesting that citalopram increases autophagy activity [143]. In a glial context, escitalopram and citalopram both exhibit anti-inflammatory effects, including reducing pro-inflammatory cytokines, such as TNF-α, IL-6, and IL-1β [144,145]. In a study, Su et al. showed that escitalopram inhibits M1 microglial polarization while promoting M2 microglial activation [146]. Citalopram can significantly increase BDNF expression and improve cell survival [147]. In addition, a study by Ishima et al. revealed that escitalopram potentiates NGF-induced neurite outgrowth through S1R activation, as a selective S1R antagonist blocked this effect [148]. Preclinical and clinical studies have demonstrated that citalopram and escitalopram are not only anti-depressive and anxiolytic in AD patients but also reduce the toxic effects of amyloid precursor protein and Aβ in AD and other amyloid-related disorders [143,149,150,151,152,153,154]. In a PD model, escitalopram and citalopram were also found to mildly inhibit neuroinflammation, dopaminergic neuronal death, and motor dysfunction [155]. Most importantly, a clinical study also reported that citalopram improved motor performance and bradykinesia in a PD patient [156]. Briefly, citalopram and escitalopram exhibit multitarget therapeutic promises by extending their benefits beyond traditional serotonergic actions. Through S1R activation, they regulate ER stress, improve autophagy, reduce neuroinflammation, and induce neuronal repair. These properties position them as promising candidates for treating neurodegenerative disorders.

### 3.3. Fluoxetine

Fluoxetine (Prozac, Fontex, and Ladose), another SSRI, is not only renowned for its efficacy in treating depression but also shows promising neuroprotective properties through activation of S1R (K_i_ = 240 nM) [157]. As reviewed by Bougea et al., fluoxetine has been shown to have some neuroprotective properties in AD models. It is thought to have the ability to reduce Aβ and improve cognitive performance [158]. Fluoxetine has been shown to enhance the activity of key mitochondrial enzymes involved in the ETC and TCA cycle, including complexes I–IV, glutamate dehydrogenase, and ATP synthase, suggesting that fluoxetine supports mitochondrial function by promoting energy production [159,160,161,162]. It has also been shown that fluoxetine inhibited ER stress in a PD model by suppressing XBP1/caspase-3-mediated ER stress and CHOP expression. In this study, fluoxetine reversed dopaminergic neuron loss and significantly improved catalepsy symptoms and enhanced locomotor activity [163]. Fluoxetine increases autophagy-associated proteins (LC3-II and ATG5) that are consistent with autophagy activation, suggesting that fluoxetine contributes to the clearance of protein aggregates and damaged organelles [164,165,166]. Research studies also indicate that fluoxetine modulates the levels of inflammatory factors, such as TNF-α, IL-1β, -2, -6, and INF-γ [167]. Fluoxetine administered prior to and during LPS (a strong pro-inflammatory stimulus) exposure prevented LPS-induced inhibition of LTP and learning deficits. The ability of fluoxetine to modulate LTP production was mimicked by a selective S1R agonist (i.e., PRE-084) [168]. Fluoxetine inhibits M1 activation and promotes M2 microglial polarization in vitro, providing further support for its anti-inflammatory properties [146]. In a study by Lee et al., fluoxetine protected oligodendrocytes from apoptosis and prevented myelin loss by inhibiting microglial activation [169]. In this regard, a pilot study on patients who had relapsing-remitting MS revealed that the ingestion of 20 mg of oral fluoxetine daily for 24 weeks reduced the formation of new lesions in the brain [170]. Fluoxetine has also been demonstrated to enhance BDNF and NGF concentrations via S1R activation [166,171,172]. As demonstrated, fluoxetine potentiates NGF-induced neurite outgrowth through S1R activation, an effect that was impaired while using an S1R antagonist [148]. In a study by Grote et al., fluoxetine was shown to rescue deficits in neurogenesis and prevent atrophy in the DG of HD mice, likely due to neurotrophic factor modulation [173]. In summary, SSRIs (such as fluvoxamine, fluoxetine, citalopram, and escitalopram), commonly recommended for altered mood disorders, now appear to have many potential benefits in relieving the symptoms of neurodegenerative diseases. Their dual mechanisms of action not only combat serotonin imbalances but also target S1R and subsequently confer broad neuroprotective effects including, but not limited to, improving autophagy, reducing neuroinflammation, stimulating neurotrophins, and modulating amyloid and tau pathologies, making them compelling candidates for repurposing in neurodegenerative disorders. As our understanding of S1R biology expands, SSRIs exemplify how repurposing existing drugs can unlock novel therapies for debilitating neurological conditions.

### 3.4. Memantine

Memantine (Namenda, Axura, and Memox)™, a cornerstone therapy for moderate to severe AD [174], exerts its neuroprotective effects through dual mechanisms: the uncompetitive antagonism of NMDA receptors and the inhibition of Aβ pathology [175,176,177]. Its affinity for NMDA receptors is approximately 0.5–1 μM, although this value varies depending on the receptor subunit composition and experimental conditions used to assess binding [178,179]. By blocking excessive glutamate-induced NMDAR activation, memantine mitigates excitotoxicity while preserving physiological synaptic transmission, which is critical for neuronal survival. In addition to its NMDA antagonism, memantine also binds to S1R, with a reported K_i_ of approximately 2.5 μM. Although this reflects relatively low affinity, S1R activation is known to modulate NMDA receptor function. Therefore, memantine’s dual mechanism—through both direct NMDA receptor antagonism and indirect modulation via S1R—may exert synergistic neuroprotective effects, which are particularly relevant for the treatment of neurodegenerative diseases [103,105,106,117]. In this context, Keshavarz et al. showed that memantine reduced the neurotoxic effects of Aβ and increased cell viability and that blocking S1R reduced the neuroprotective effects of memantine [180]. It has been indicated that memantine enhances the activity of mitochondrial ETC enzymes and supports mitochondrial membrane potential. Additionally, it could reduce oxidative stress parameters, such as NO levels and SOD activity. Memantine also increases the expression of cell survival genes, including CREB and BDNF [181]. Furthermore, memantine treatment improves the inflammatory cytokine profile by reducing IL-6 and TNF-α levels [182]. In support of the role of memantine in neurodegenerative diseases, treatment with memantine in PD patients has been associated with lower scores for axial motor symptoms and dyskinesia [183]. Trials have also examined memantine’s effectiveness and safety in treating PD-related dementia and found it to be well-tolerated and effective [184,185]. A pilot study in HD patients showed that 20 mg of memantine once a day significantly improved motor symptoms, primarily by alleviating chorea, without causing significant side effects [186]. Memantine exemplifies a nuanced approach to neurodegeneration, balancing NMDA receptor antagonism with S1R-mediated neuroprotection. By addressing excitotoxicity, oxidative stress, and inflammation, it holds promise not only in AD but also in PD and HD, advocating for repurposing efforts to maximize its clinical potential.

### 3.5. Dextromethorphan

Dextromethorphan (Robitussin, Delsym, and Mucinex DM)™ is widely recognized as an antitussive. Beyond its cough-suppressing role, it acts as a non-competitive antagonist of NMDA receptors, with a binding affinity in the range of 0.5 to 2 µM, and also modulates voltage-gated calcium channels [187,188,189]. Dextromethorphan is also a potent S1R agonist, with a binding affinity (K_i_) ranging between 142 and 652 nM. Its neuroprotective effects are likely mediated, at least in part, through its binding to S1R [190,191]. Interestingly, dextromethorphan has been shown to be beneficial for treating levodopa-induced dyskinesia in patients with PD [192]. For a long time, it was assumed that dextromethorphan’s effects in PD were mediated via NMDA receptor antagonism. In a PD model, Paquette et al. showed that dextromethorphan suppressed levodopa-induced dyskinesia, while other NMDA antagonists failed to suppress dyskinesias. Notably, the S1R antagonist could reverse the effects of dextromethorphan, suggesting that S1R activity plays a crucial role in mediating its therapeutic effects [193]. Dextromethorphan inhibits the release of pro-inflammatory cytokines, such as TNF-α, from microglial cells [194,195]. It also blocked microglial activation and degeneration of dopaminergic neurons in LPS-treated cultures [196,197]. In fact, Zhang et al. showed that dextromethorphan significantly reduced the MPTP-induced production of extracellular superoxide radicals and ROS by modulating NADPH oxidase [198]. Long-term treatment with dextromethorphan in experimental animal models of MS showed a reduction in neutrophil infiltration by the inhibition of NADPH oxidase 2 and attenuated axonal demyelination by the upregulation of S1R [199]. These mechanisms may also underline dextromethorphan’s potential utility in managing complex disorders, where neuroinflammation is dominant. Dextromethorphan’s rapid metabolism to dextrorphan (via CYP2D6) limits bioavailability. Co-administration with quinidine (a CYP2D6 inhibitor) or bupropion enhances plasma concentrations, unlocking its neuroprotective potential. Combined with bupropion hydrochloride (Auvelity)™, it is now FDA-approved for the treatment of major depressive disorder in adults [200,201]. It is also important to note that dextromethorphan most prominently binds in the brainstem and cerebellum, areas particularly rich in S1R, and is implicated in pseudobulbar affects (PBAs) [202]. In this context, several clinical trials have demonstrated that dextromethorphan, particularly when combined with quinidine, helps alleviate symptoms of PBAs (NCT01806857, NCT03883581, and NCT00573443). This combination, marketed as Nuedexta™, is FDA-approved and indicated for the treatment of PBAs in patients diagnosed with ALS and MS [203,204,205,206]. Additionally, Fox et al. demonstrated that dextromethorphan/quinidine (45 mg/10 mg twice daily) was well tolerated and significantly reduced dyskinesia severity in patients with PD undergoing treatment for levodopa-induced dyskinesia [207]. Furthermore, the compound demonstrated its efficacy during clinical trials for AD and PD dementia, making it more promising when it comes to degenerative diseases [208,209]. In conclusion, dextromethorphan clinical repurposing in formulations like Auvelity™ and Nuedexta™ underscores the value of combinatorial approaches to overcome metabolic limitations. As research unravels its full potential, dextromethorphan stands poised to redefine treatment paradigms in PD, MS, and beyond, offering hope for conditions where neuroinflammation and excitotoxicity converge.

### 3.6. Amantadine

Amantadine (Symmetrel, Gocovri, and Dyskinesia)™ was originally developed as an antiviral agent and was later recognized as an NMDA receptor antagonist. It exhibits moderate binding affinity for NMDA receptors, with reported K_i_ values ranging from approximately 10 to 50 μM, depending on assay conditions and receptor subtypes [179,210]. Amantadine has been used as a long-term treatment for PD and is currently prescribed in approximately 10% of patients, either as monotherapy or in combination with L-DOPA [211]. However, its precise mechanism of action in PD remains incompletely understood. In a clinical study by Uitti et al., amantadine use was identified as an independent predictor of improved survival in patients with parkinsonism. The authors suggest that this effect may result from either ongoing symptomatic benefits or a direct neuroprotective mechanism, possibly mediated through NMDA receptor antagonism or dopamine reuptake inhibition [212]. Additionally, Peeters et al. demonstrated that amantadine dose-dependently reduced the specific binding of a selective S1R radioligand in the striatum, indicating a physiologically relevant interaction with the S1R [117]. Amantadine is considered a class of drugs that increases dopamine levels and is therefore beneficial in the treatment of depression and PD [210,213]. Importantly, Peeters et al. demonstrated that the modulatory effects of amantadine on dopaminergic transmission were abolished when S1R was antagonized, further implicating S1R in its mechanism of action [117]. In addition to its dopaminergic effects, it has been reported that amantadine decreases oxidative stress and neuroinflammation, which may further support its neuroprotective role in neurodegenerative diseases [214,215]. In PD, immediate-release amantadine is typically administered two to four times daily. However, its therapeutics are relatively short-lived, which can result in fluctuations in symptom control throughout the day [207]. To address this limitation, extended-release amantadine formulations—currently approved by the U.S. Food and Drug Administration—have been developed. These formulations offer more consistent plasma levels and have demonstrated efficacy in reducing motor symptoms such as tremors, rigidity, and bradykinesia during both dyskinesia and OFF episodes [216,217]. In addition to PD, in a randomized, controlled trial, amantadine also effectively reduced chorea severity in HD patients without significant cognitive impairment or worsening of parkinsonism [218].

### 3.7. Donepezil

Donepezil (Aricept, Donecept, and Episenta)™ is a widely prescribed acetylcholinesterase inhibitor with a binding affinity of 0.65–2.3 μg/g in rodents (in µg drug per gram brain weight) [219], approved by the FDA for treating AD. While its primary mechanism involves increasing acetylcholine levels in the brain and improving cognitive abilities, emerging evidence highlights its broader therapeutic potential, extending beyond its cholinergic effects [220,221,222]. According to the findings of Ramakrishnan et al., with a high binding affinity (K_i_) of 14.6 nM for the S1R, donepezil achieves 90% occupancy of the S1Rs in the brain and acts as an S1R agonist, thus extending its effects to additional neuroprotective mechanisms [223]. The administration of donepezil has been shown to enhance several mitochondrial functions, including basal respiration, maximal respiration, and ATP production. Notably, donepezil also induced an increase in mitochondrial membrane potential, which supports the notion that it enhances mitochondrial health and bioenergetics [224,225]. It has been shown that donepezil upregulates pAMPK and LC3 II/I and downregulates p70S6K and p-p62 (Ser351) levels, which are key regulators of autophagy [226]. In animal studies, donepezil reduces brain Aβ accumulation, and its neuroprotective effect against Aβ appears to involve direct interactions with the S1R [227,228]. Solntseva et al. showed that donepezil antagonized the suppressive effects of Aβ on LTP, and an S1R agonist (PRE-084) mimicked the effects of donepezil and the S1R antagonist (haloperidol), reversing it [229]. It has been also demonstrated that donepezil decreased the Aβ-induced increase in ROS [31]. Interestingly, donepezil ameliorates PCP-induced cognitive deficits in mice. The effect of donepezil on these deficits was significantly attenuated by the co-administration of the selective S1R antagonist, suggesting involvement of the S1 receptor in donepezil’s mechanisms of action [230]. Furthermore, donepezil has been shown to significantly potentiate NGF-induced neurite outgrowth, a process not observed with another acetylcholinesterase inhibitor, physostigmine. This effect of donepezil was blocked by the co-administration of a selective S1R antagonist or an IP3 receptor antagonist, again suggesting the critical role of S1R and its interaction with IP3 receptors in the pharmacological action of donepezil [231].

A new class of compounds has emerged as a cutting-edge strategy for addressing AD and related neurodegenerative disorders, namely, donepezil–flavonoid hybrid compounds (DFHs). These hybrids synergize the neuroprotective properties of flavonoids with the acetylcholinesterase inhibitory activity of donepezil while also targeting S1R and other key pathological pathways, including 5-Lipoxygenase and monoamine oxidases. [232]. Donepezil may also help reduce neuroinflammation while improving neuronal survival and protection against excitotoxicity. In ALS models, nebivolol–donepezil hybrids were able to reduce the level of the cytokine secreted by microglial cells, inhibit the translocation of NF-κB into the nucleus, and protect against excitotoxicity by acting through the PI3K–Akt pathway [233]. Donepezil–flavonoid hybrids represent a paradigm shift in neurodegenerative disease treatment, merging the cognitive benefits of acetylcholinesterase inhibition with flavonoid-derived antioxidant, anti-inflammatory, and S1R-mediated neuroprotection. By targeting multiple pathways—cholinergic deficits, protein aggregation, neuroinflammation, and oxidative stress—DFHs offer a holistic strategy to slow disease progression and alleviate symptoms. Their development underscores the growing emphasis on multitarget therapies to address the complex pathology of AD, ALS, and other neurodegenerative disorders, positioning DFHs as a transformative avenue for future drug discovery.

## 4. Conclusions

Collectively, this information highlights the utility of repurposing existing compounds to address the complex pathology of neurodegenerative diseases. By targeting mechanisms such as mitochondrial dysfunction, protein aggregation, oxidative stress, and neuroinflammation—common across AD, PD, HD, ALS, and MS—they offer multifaceted therapeutic benefits. Interestingly, S1R agonism emerges as a unifying mechanism that amplifies intracellular signaling to promote neuronal survival and plasticity while mitigating disease progression. Investigational S1R agonists like pridopidine, which have demonstrated potential in clinical trials for neurodegenerative disorders, further support the therapeutic relevance of targeting S1R [234]. However, additional research is required to evaluate the long-term safety and efficacy of sustained S1R modulation, particularly in aged or vulnerable nervous systems. To date, most available clinical studies have been limited by small sample sizes, short follow-up periods, and insufficiently rigorous control conditions. Comprehensive preclinical investigations, along with long-term, well-powered, and methodologically robust clinical trials, are essential to fully characterize the therapeutic potential and safety profile of S1R-targeting compounds.

Drug repurposing strategies, while promising, face several important translational and regulatory challenges. One major issue is the uncertainty surrounding optimal dosing for new indications. For example, in PD patients, some studies suggest that SSRIs may potentially worsen motor symptoms or contribute to drug-induced parkinsonism [235], whereas others report no such effects [156,236,237,238,239]. Given this variability, careful patient monitoring and individualized dose adjustments are essential. Additional barriers include the lack of intellectual property protection for off-patent compounds, limited commercial incentives—especially when generic formulations are available—and complexities related to pricing and reimbursement further, which complicate the clinical development of repurposed therapies [240]. Nonetheless, drug repurposing remains a cost-effective and time-efficient strategy for advancing therapies for neurodegenerative diseases. By leveraging existing data on safety, pharmacokinetics, and tolerability and through coordinated efforts among scientific, regulatory, and commercial sectors, this strategy holds significant potential to expedite the delivery of effective treatments to patients in need.

## Figures and Tables

**Table 1:** Potential repurposed drugs targeting S1R.

Drug Name	2D Structure	Primary Target MOA	Currently Approved	Radioligand S1R K_i_	S1R MOA	Potential Repurposing Disease(s)
Fluvoxamine	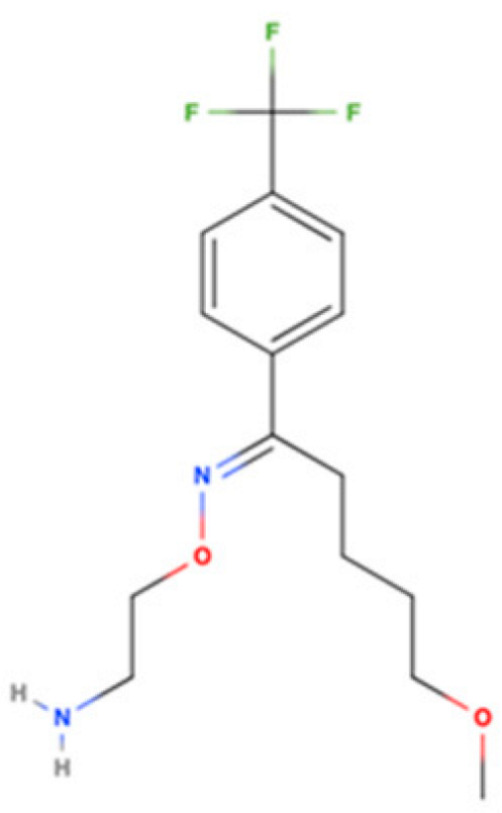	SSRI	Psychiatric disorders (e.g., depression and obsessive-compulsive disorder)	[3H](+)-pentazocine 36 nM [116]	Agonist neuroprotection (↑calcium influx into mitochondria) ↓γ-secretase activity ↓ER stress ↑autophagy ↓neuroinflammation ↑neurotrophic factors like BDNF and NGF	Cognitive impairments, AD, MS, ALS, and HD
Citalopram	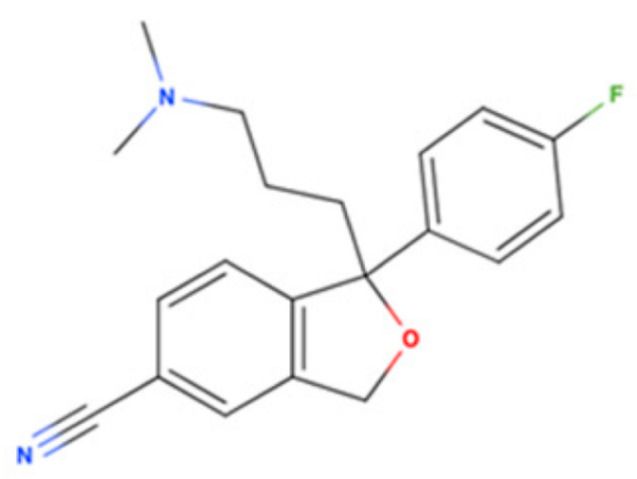	SSRI	Psychiatric disorders (e.g., depression and obsessive-compulsive disorder)	[3H](+)-pentazocine 292 nM [116]	Agonist ↓pro-inflammatory cytokines (TNF-α, IL-6, and IL-1β) ↑autophagy ↓mutant amyloid precursor protein (APP) and Aβ ↑neurotrophins like BDNF	Amyloid-related disorders (e.g., AD) and movement disorders (e.g., PD)
Escitalopram	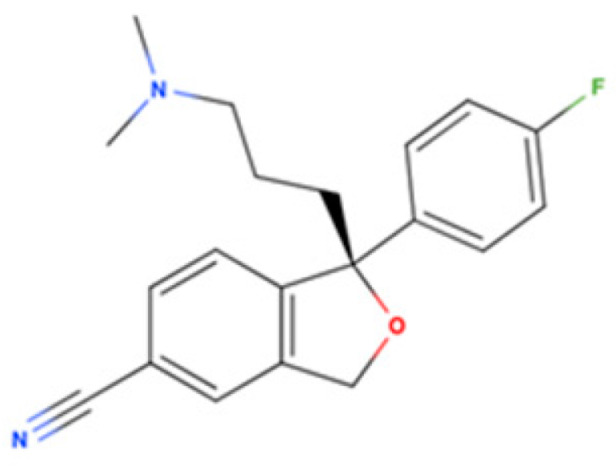	SSRI	Psychiatric disorders (e.g., depression and obsessive-compulsive disorder)	[3H](+)-pentazocine 288.3 nM [116]	Agonist ↓ER stress ↑autophagy ↑M2 microglial activation ↓neuroinflammatory response ↓Aβ burden ↑NGF-induced neurite outgrowth	Amyloid-related disorders (e.g., AD) and movement disorders (e.g., PD)
Fluoxetine	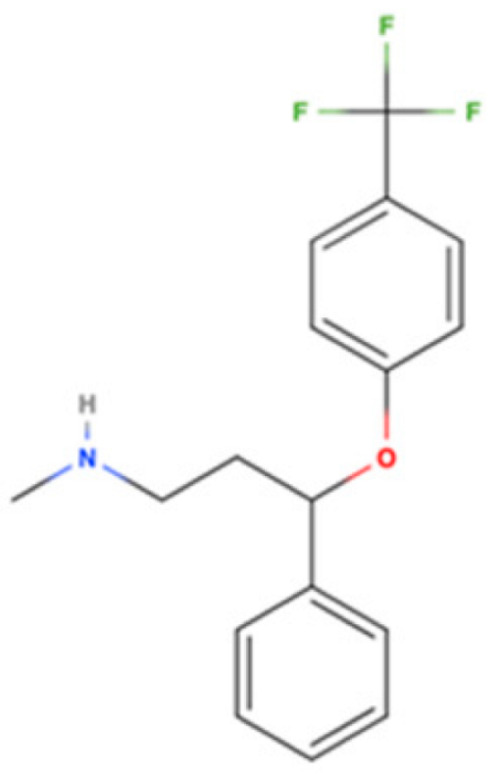	SSRI	Psychiatric disorders (e.g., depression and obsessive-compulsive disorder)	[3H](+)-pentazocine 240 nM [116]	Agonist ↑ATP by increasing mitochondrial function ↓ER-stress ↓pro-inflammatory cytokines (TNF-α, IL-1β, and INF-γ) regulate microglial activation (↓M1 and ↑M2 activity) ↑autophagy ↑BDNF and NGF	AD, PD, and HD
Memantine	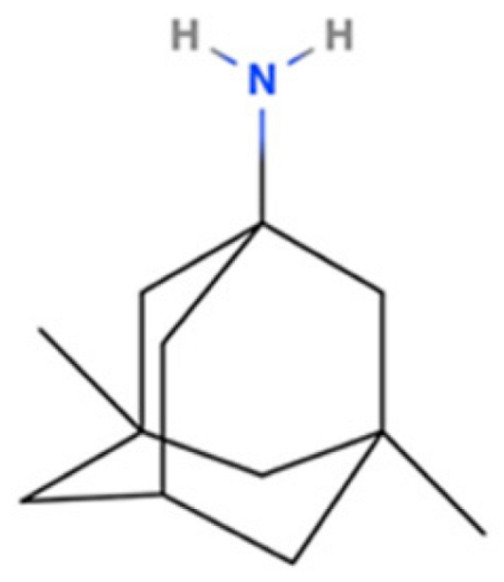	NMDA Receptor Antagonist	AD	[3H]-(+)SKF-10,047 2.5 μM [117]	Agonist ↑mitochondrial function ↓oxidative stress ↓inflammatory cytokines (TNF-α and IL-6) ↓Aβ production ↑BDNF	Neurodegene-rative disorders (e.g., PD and HD)
DXM	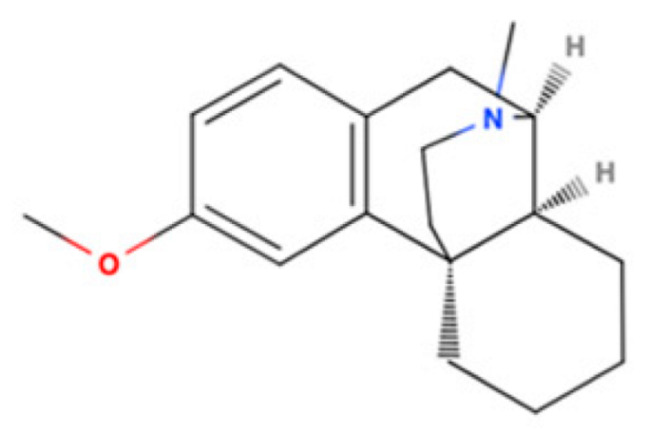	NMDAR Antagonist	Cough suppressant and pseudobulbar effect in combination with quinidine	[3H]-(+)SKF-10,047 205 nM [118]	Agonist ↓neuroinflammation ↓ER stress ↓ROS generation ↑NGF	PD, MS, and ALS
Amantadine	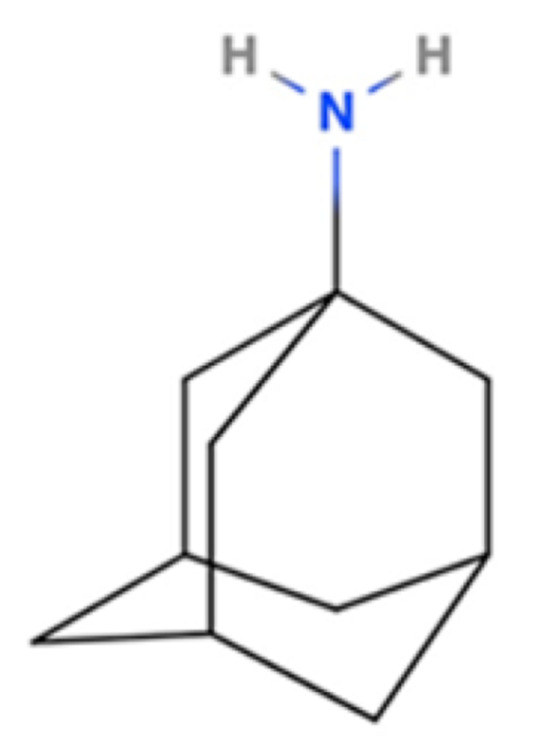	NMDA Receptor Antagonist	PD	[3H]-(+)SKF-10,047 7 μM [117]	Agonist ↑dopaminergic transmission ↓oxidative stress ↓neuroinflammation	Movement disorders (e.g., HD)
Donepezil	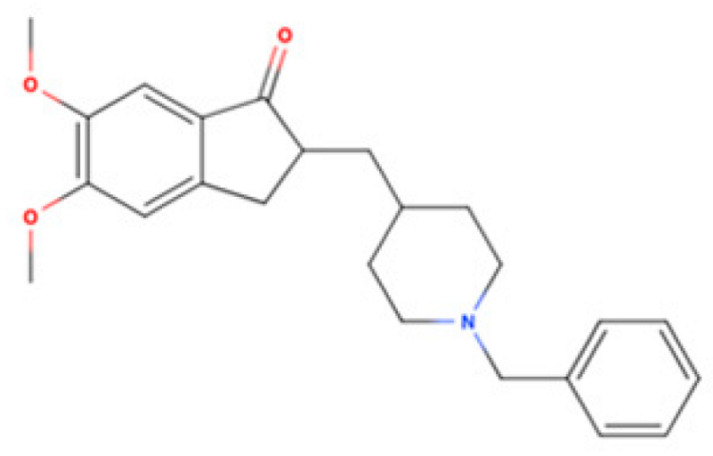	AChE Inhibitor	AD	[3H]DTG 14.6 nM [119]	Agonist ↑mitochondrial function ↓Aβ accumulation ↓neuroinflammatory responses ↓excitotoxicity ↑NGF	Cognitive deficits, ALS, and other neurodegene- rative disorders

MOA, mechanism of action. DXM, dextromethorphan; AChE, acetylcholinesterase.

## Data Availability

No original data was generated or analyzed in the preparation of this review article. Data sharing is therefore not applicable. All figures are covered under a BioRender Academic Publication License.

## Abbreviations

The following abbreviations are used in this manuscript:

AD	Alzheimer’s disease
PD	Parkinson’s disease
HD	Huntington’s disease
ALS	Amyotrophic lateral sclerosis
MS	Multiple sclerosis
Aβ	β-amyloid
TDP-43	TAR DNA-binding protein 43
S1R	Sigma-1 receptor
ER	Endoplasmic reticulum
MAM	Mitochondria-associated membrane
BiP	Binding immunoglobulin protein
IP3R	Inositol triphosphate receptor
PLC	Phospholipase C
TCA	Tricarboxylic acid cycle
ETC	Electron transport chain
ROS	Reactive oxygen species
Rac1	Ras-related C3 botulinum toxin substrate 1
VDAC2	Voltage-dependent anion channel 2
NQO1	NAD(P)H quinone oxidoreductase 1
SOD1	Superoxide dismutase 1
XBP1	X-box-binding protein 1
CHOP	C/EBP homologous protein
BCL-2	B-cell lymphoma 2
BAX	BCL-2-associated X protein
BAK	BCL-2 antagonist/killer
ULK	Unc-51-like autophagy-activating kinase
mTORC1	Mammalian target of rapamycin complex 1
AMPK	AMP-activated protein kinase
TFEB	Transcription factor EB
BDNF	Brain-derived neurotrophic factor
NGF	Nerve growth factor
GDNF	Glial cell-derived neurotrophic factor
EGF	Epidermal growth factor
TrkB	Tropomyosin receptor kinase B
CaMKIV/II	Calcium/calmodulin-dependent protein kinases IV/II
CREB	cAMP response element-binding protein
NMDA	*N*-Methyl-D-aspartate
GluN2A/B	Glutamate receptor subunits 2A/B
JAK2	Janus kinase 2
STAT3	Signal transducer and activator of transcription 3
GFAP	Glial fibrillary acidic protein
ERK	Extracellular signal-regulated kinase
NF-κB	Nuclear factor kappa B
TLR4	Toll-like receptor 4
SSRI	Selective serotonin reuptake inhibitor
LTP	Long-term potentiation
CYP2D6	Cytochrome P450 2D6
